# Review of Outcomes Between Robotic and Thoracoscopic Thoracic Surgery: A Retrospective Cohort Study

**DOI:** 10.7759/cureus.86432

**Published:** 2025-06-20

**Authors:** Amira Barmanwalla, Sriram Rangarajan, Aaron Fowler, Gunjan Bhat, Harpreet Gill, Aldin Malkoc, Angel Guan, Amanda Daoud, Rafael A Navarro, Lawrence B Kong

**Affiliations:** 1 General Surgery, Arrowhead Regional Medical Center, Colton, USA; 2 Thoracic Surgery, Kaiser Permanente, Fontana, USA

**Keywords:** lung adenocarcinoma, lung cancer, robotic, robotic-assisted thoracoscopic surgery (rats), robotic-assited surgery, video-assisted thoracoscopic surgery (vats)

## Abstract

Background and aims: Surgical management of lung cancer has evolved over the last two decades from open thoracotomy to video-assisted thoracoscopic surgery (VATS) and, more recently, robotic-assisted thoracoscopic surgery (RATS). We present our initial experience with RATS and hypothesize that overall outcomes will be improved compared to VATS and will result in overall cost savings.

Methods: A retrospective review of prospectively collected data was conducted on all patients who underwent RATS over the first year and a half since robotic surgery was introduced, compared to VATS over the two years prior to the initiation of robotic surgery in our institution. Demographics, operative, and postoperative data were obtained, and statistical analysis was performed using a t-test.

Results: Patients undergoing RATS (n = 42) were older (70.31 +/- 8.89 vs. 64.64 +/- 12.53, p = 0.018) compared to the VATS group (n = 48). RATS operative times were longer, 190.26 minutes compared to 127.84 minutes (P=0.001) in the VATS group. Estimated blood loss (EBL) was significantly lower in the RATS group (74 ml versus 208 ml; p < 0.05). The mean length of stay was shorter in the RATS group (3.88 versus 6.22 days; p < 0.05). All postoperative outcomes were similar, including complications and 30-day mortality (RATS, 2.1%, versus VATS, 4.2%; p > 0.05). There were no statistical differences in length of intensive care unit (ICU) stay (0.3 versus 1.2 days; p > 0.05) or number of lymph nodes harvested (4.21 versus 3.81; p > 0.05).

Conclusion: RATS is safe and technically feasible, and short-term outcomes are comparable to those of the conventional VATS approach.

## Introduction

Early-stage primary lung carcinomas, typically stage I and II, are initially treated with surgical resection. Standard surgical guidelines recommend removing the affected lobe along with ipsilateral hilar and mediastinal lymph nodes. The primary goal is to reduce morbidity and mortality using minimally invasive techniques that preserve lung volume and function while ensuring adequate lymph node retrieval for accurate staging. Following resection, pathologic staging is performed to determine the need for additional treatment [1].

Advancements in surgical techniques and technology have transformed thoracic surgery, improving patient outcomes and procedural precision [2]. Among these innovations, two leading minimally invasive approaches have emerged: robotic-assisted thoracic surgery (RATS) and video-assisted thoracic surgery (VATS) [3]. RATS has gained attention for its ability to address limitations of open thoracotomy and conventional VATS [4]. Both RATS and VATS employ small incisions and video guidance to minimize tissue trauma and promote faster recovery [5]. However, RATS offers advantages such as enhanced dexterity, precision, and visualization, enabling complex maneuvers within the confined thoracic cavity. The limitations include the absence of haptic sensation, higher costs, prolonged operative times, and difficulty in the learning curve for the operative team [6].

Comparing RATS and VATS is clinically significant, as it can inform surgical decision-making and support evidence-based practices. Evaluating operative outcomes, including postoperative pain, hospital length of stay, complications, oncologic staging (e.g., lymph node harvest), and patient satisfaction, helps highlight the strengths and limitations of each technique. This study aims to compare the operative outcomes of RATS and VATS.

## Materials and methods

A retrospective observational cohort review of patients who underwent RATS or VATS at a single high-capacity quaternary center between January 2015 and December 2020 was performed. Information was gathered about all patients admitted to the hospital with operative lung cancer. Exclusion criteria included patients under the age of 18 years. For all 87 patients identified, a retrospective chart review was performed to obtain admission records and inpatient records. Data gathering and analysis were conducted according to the Kaiser Permanente Institutional Review Board (IRB), Fontana, CA, USA (IRB #10448). Consent was obtained prior to starting the study.

The data collected included patient age, gender, medical comorbidities, and postoperative complications. Preoperatively, patients underwent risk stratification based on comorbidities with cardiac workup and pulmonary function testing. Forced expiratory volume in one second (FEV1)/forced vital capacity (FVC) ratios and diffusing capacity of the lungs for carbon monoxide, adjusted for alveolar volume (DLCO-Va), are both key parameters in pulmonary function tests that assess lung function and gas exchange. The primary operative endpoints included operative time (minutes), blood loss (mL), and intraoperative transfusion requirements (units of packed red blood cells (pRBC)). The primary postoperative outcomes included length of stay (days), ICU stay (days), 30-day complications, and number of lymph nodes harvested. A broad range of thoracic diseases was looked at, with the majority being lung cancer, as well as some benign lung lesions, single metastases, thymic lesions, and nodular fibrosis. The RATS and VATS groups included wedge resections, segmentectomies, and lobectomies.

The data were collected using Microsoft Excel (Microsoft Corp., Redmond, WA, USA), and the data were analyzed using IBM SPSS Statistics software, version 27.0 (IBM Corp., Armonk, NY, USA). Continuous data are presented according to the means with standard deviation, and categorical data are presented with frequencies and proportions. A t-test was utilized for continuous data, and where appropriate, non-parametric continuous data was analyzed using Mann-Whitney U tests. Univariate analyses were performed using chi-squared tests for categorical data. Propensity score-matched analysis was performed to create comparable groups in the RATS and VATS cohorts. Pairs were formed such that the matched subjects had similar propensity score values, and a caliper distance of 0.1 was used. The patients were matched for age, sex, type of pulmonary pathology, and pathologic stage. A multivariate analysis was performed, and a Kaplan-Meier curve was generated while adjusting for other risk factors. Unless otherwise indicated, a p-value less than 0.05 was statistically significant.

## Results

**Figure 1:**
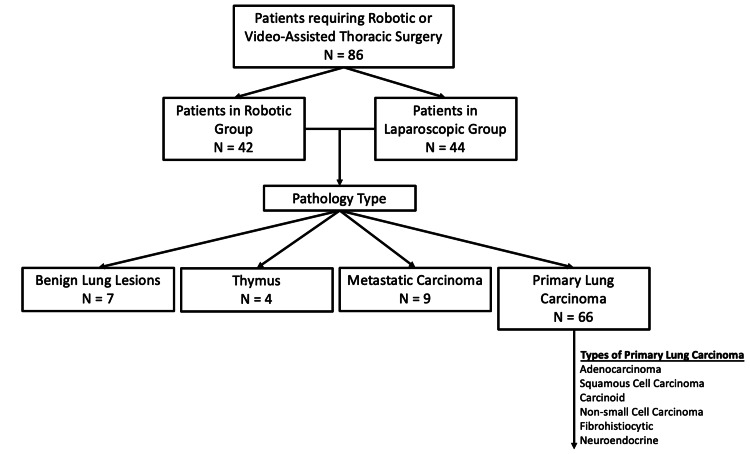
A Consolidated Standards of Reporting Trials (CONSORT) diagram detailing the patients who had either robotic or video-assisted thoracic surgery and the types of pathologies.

From the 86 patients, there were a total of 42 patients in the RATS group and 44 patients in the VATS group. The Consolidated Standards of Reporting Trials (CONSORT) diagram in Figure 1 summarizes the details, including the types of pathologies. In the study, the mean age for RATS was 70.31 ± 8.89 and for VATS 64.64 ± 12.53 (p=0.018). There was no significant difference in individual comorbidities. Among the total number of comorbidities, RATS had a mean of 2.25 ± 1.27, and VATS was 1.57 ± 1.50 (p=0.027). Total hospitalization stay was significantly shorter in the RATS group compared to the VATS group (RATS, 3.88 ± 3.26; VATS, 6.22 ± 5.82 (p=0.023)). There were no significant differences when considering intensive care unit (ICU) length of stay, postoperative complications, or hospital mortality. The remainder of the results are summarized in Table 1. 

**Table 1:** Baseline patient characteristics with total patient population and propensity matched analysis ^a^ analyzed using the non-parametric Mann-Whitney U test; (*) representing a significant p-value

Parameters	Total patient population			Propensity matched analysis for age, gender, tumor type, and pathologic stage		
Demographics	Video-assisted thoracoscopic surgery N = 44	Robotic-assisted thoracoscopic surgery N = 42	P	Video-assisted thoracoscopic surgery N = 14	Robotic-assisted thoracoscopic surgery N = 14	P
^a^Age	70.31 ± 8.89	64.64 ± 12.53	0.018*	68.85 ± 11.22	65.78 ± 17.50	0.586
Gender						
Male	17 (38.6%)	29 (69%)	0.005*	8 (57.1%)	7 (50%)	0.705
Female	27 (61.4%)	13 (31%)		6 (42.9%)	7 (50%)	
Comorbid conditions						
Diabetes mellitus (%)	16 (36.4%)	13 (31%)	0.596	7 (50%)	4 (28.6%)	0.246
Hypertension (%)	30 (68.2%)	24 (57.1%)	0.290	10 (71.4%)	6 (42.9%)	0.127
Hyperlipidemia (%)	23 (52.3%)	4 (9.5%)	0.001*	8 (57.1%)	2 (14.3%)	0.018*
Chronic lung disease (%)	10 (22.7%)	8 (19%)	0.675	2 (14.3%)	4 (28.6%)	0.357
Coronary artery disease (%)	8 (18.2%)	9 (21.4%)	0.705	4 (28.6%)	5 (35.7%)	0.686
Chronic kidney disease (%)	5 (11.4%)	4 (9.5%)	0.781	1 (7.1%)	3 (21.4%)	0.280
Atrial fibrillation %	7 (15.9%)	4 (9.5%)	0.375	3 (21.4%)	2 (14.3%)	0.622
Total comorbidities	2.25 ± 1.27	1.57 ± 1.50	0.027*	2.5 ± 1.40	1.85 ± 1.87	0.314
Hospital length of stay	6.22 ± 5.82	3.88 ± 3.26	0.023*	5.07 ± 2.05	4.50 ± 3.52	0.606
Intensive care unit length of stay	1 ± 5.1	0.69 ± 2.7	0.727	0.0	1.07 ± 3.73	0.302
Complications %	8 (18.2%)	11 (26.2%)	0.371	3 (21.4%)	4 (28.6%)	0.663
Hospitalization mortality						
Alive (%)	42 (95.5%)	39 (92.9%)	0.607	14 (100%)	12 (85.7%)	0.142

FEV1/FVC ratios for the VATS group were 69.82 ± 12.25 and for RATS were 86.68 ± 19.39 (p=0.001). Among the carcinomas, 91% of the VATS group and 62% of the RATS group had primary lung adenocarcinoma. Notably, lung adenocarcinoma was the most common pathology among both groups. The remainder of the results are summarized in Table 2.

**Table 2:** Preoperative patient pulmonary characteristics with total patient population and propensity-matched analysis (*) representing a significant p-value FEV1/FVC: forced expiratory volume in one second/forced vital capacity; DLCO-Va: diffusing capacity of the lungs for carbon monoxide, adjusted for alveolar volume

Parameters	Total patient population			Propensity matched analysis for age, gender, tumor type, and pathologic stage		
Measures	Video-assisted thoracoscopic surgery (N = 44)	Robotic-assisted thoracoscopic surgery (N = 42)	P	Video-assisted thoracoscopic surgery (N = 14)	Robotic-assisted thoracoscopic surgery (N = 14)	P
FEV1/FVC	69.82 ± 12.25	86.68 ± 19.39	0.001*	71.06 ± 12.96	80.50 ± 12.49	0.116*
DLCO_Va	101.87 ± 21.98	84.31 ± 48.75	0.105	108.67 ± 12.89	57.91 ±​​​​​​​ 60.17	0.036
Type of cancer						
Benign	1 (2.3%)	6 (14.3%)	0.009*	0 (0%)	0 (0%)	0.622*
Thymus	0 (0%)	4 (9.5%)		0 (0%)	0 (0%)	
Metastatic carcinoma	3 (6.8%)	6 (14.3%)		3 (21.4%)	2 (14.3%)	
Primary lung carcinoma	40 (90.9%)	26 (61.9%)		11 (78.6%)	12 (85.7%)	
Subtypes of lung carcinoma						
Lung adenocarcinoma	25 (56.8%)	16 (38.1%)	0.064	8 (57.1%)	9 (64.3%)	0.515
Lung squamous cell carcinoma	8 (18.2%)	6 (14.3%)		3 (21.4%)	1 (7.1%)	
Lung carcinoid	3 (6.8%)	1 (2.4%)		0 (0%)	1 (7.1%)	
Lung sarcoma	1 (2.3%)	2 (4.8%)		0 (0%)	1 (7.1%)	
Lung non-small cell carcinoma	2 (4.5%)	0 (0%)		0 (0%)	0 (0%)	
Lung fibrohistiocytic	0 (0%)	1 (2.4%)		0 (0%)	0 (0%)	
Lung neuroendocrine and clear cell	1 (2.3%)	0 (0%)		0 (0%)	0 (0%)	

Within the VATS group, there was a noted decreased operative time of 127.84 +/- 49.13 compared to 190.26 +/- 71.88 (P=0.001). The VATS group additionally had increased blood loss of 208.97 ml +/- 240.58 ml compared to RATS 72.64 ml +/- 218.2 ml (p=0.007). There was no difference in the need for intraoperative blood transfusions. Both the VATS and RATS groups had similar tumor sizes, respectively 3.69 cm +/- 2.51 cm and 3.09 cm +/- 2.33 cm (p=0.275). The remainder of Table 3 details final tumor pathology staging. Lastly, there was no significant difference in the number of lymph nodes collected and positive lymph nodes between the VATS and RATS groups.

**Table 3:** Operative patient pulmonary characteristics along with pulmonary lesion characteristics with total patient population and propensity matched analysis (*) representing a significant p-value

Parameters	Total patient population			Propensity matched analysis for age, gender, tumor type, and pathologic stage		
Measures	Video-assisted thoracoscopic surgery (N = 44)	Robotic-assisted thoracoscopic surgery (N = 42)	P	Video-assisted thoracoscopic surgery (N = 14)	Robotic-assisted thoracoscopic surgery (N = 14)	P
Operative time (minutes)	127.84 ± 49.13	190.26 ± 71.88	0.001*	112.64 ± 37.64	201.57 ± 87.87	0.003*
Blood loss (mLs)	208.97 ± 240.58	72.64 ± 218.2	0.007*	151.42 ± 187.60	164.28 ± 365.30	0.908*
Blood transfusion (%)	4 (9.1%)	1 (2.4%)	0.184	1 (7.1%)	1 (7.1%)	1.00
Pathology						
Tumor size (cm)	3.69 ± 2.51	3.09 ± 2.33	0.275	3.03 ± 1.90	2.54 ± 2.0	0.533
Pathologic stage						
IA	12 (27.3%)	11 (26.2%)	0.064	7 (50%)	5 (35.7%)	0.840
IB	1 (2.3%)	6 (14.3%)		1 (7.1%)	2 (14.3%)	
IIA	22 (50%)	1 (2.4%)		1 (7.1%)	1 (7.1%)	
IIB	3 (6.8%)	3 (7.1%)		2 (14.3%)	3 (21.4%)	
IIIA	2 (4.5%)	2 (4.8%)		0 (0%)	1 (7.1%)	
IV	3 (6.8%)	9 (21.4%)		3 (21.4%)	2 (14.3%)	
Total lymph nodes acquired	3.81 ± 2.63	4.21 ± 4.17	0.599	3.64 ± 3.49	4.71 ± 3.40	0.419
Total lymph nodes positive	0.25 ± 0.62	0.11 ± 0.39	0.229	0.42 ± 0.85	0.21 ± 0.57	0.444

**Figure 2:**
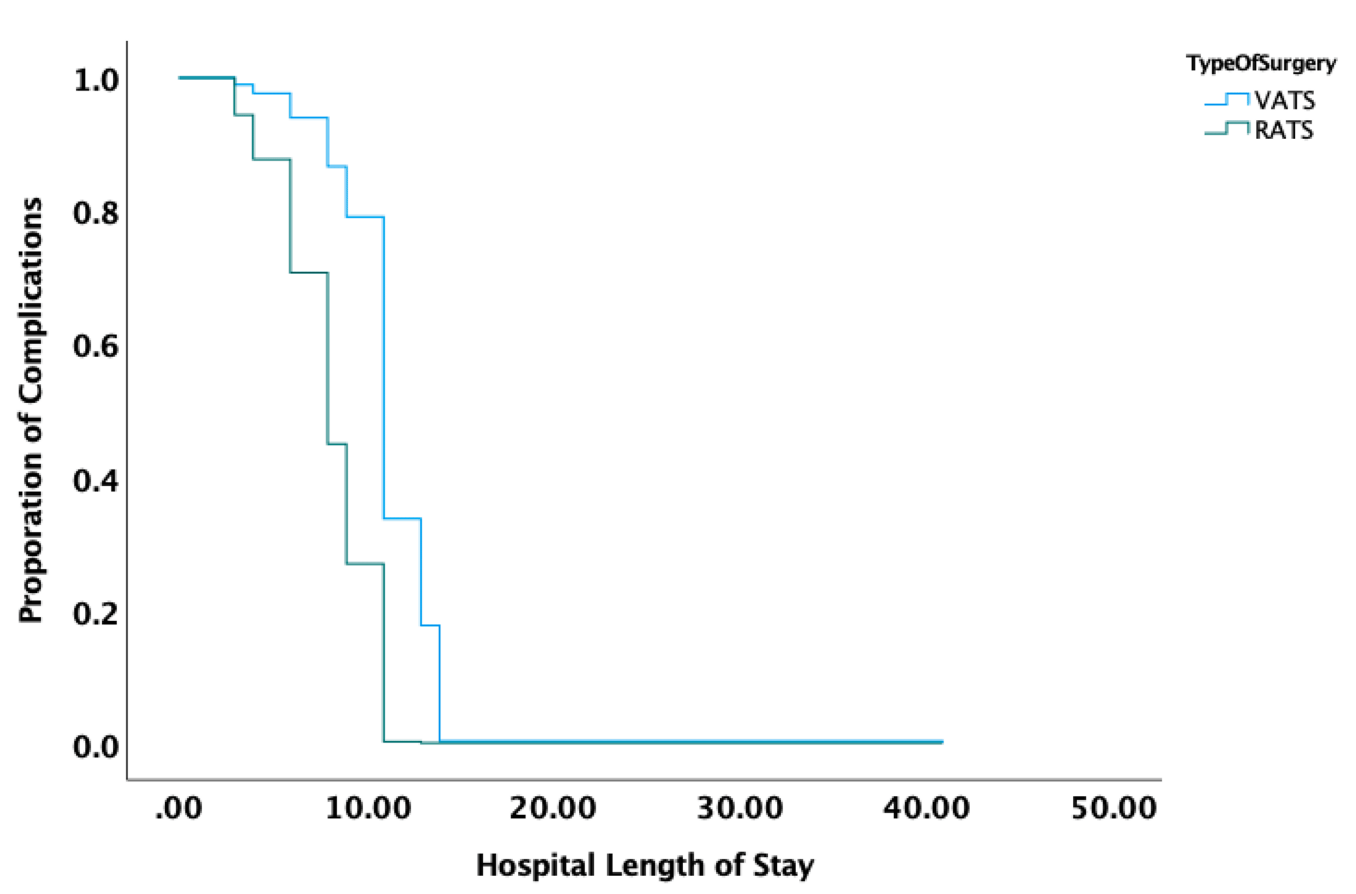
Kaplan-Meier survival probability based on either robotic or thoracoscopic surgery, demonstrating a higher risk of complications in the overall patient cohort for robotic surgery, with no statistical difference when split between type of surgery (P=0.056). VATS: video-assisted thoracoscopic surgery; RATS: robotic-assisted thoracoscopic surgery

We utilized a Kaplan-Meier analysis to model events over time, measured in patient hospitalization days. The Kaplan-Meier curve takes into consideration the total length of hospital stay and any complication event that occurred. When considering covariates of age, gender, type of surgery, pathologic tumor size, pathologic stage, operative time, and blood loss, only pathologic tumor size had a significant hazard ratio of 0.479 (p=0.023), seen in Figure 2. By day 7, there was an approximately 70% chance of a complication being present in the RATS group and an approximately 90% chance of a complication being present in the VATS group.

The propensity-matched analyses within the study in Tables 1-3 are presented alongside the total data. After propensity matching, the VATS group had 14 patients, and the RATS group had 14 patients. The VATS group had a mean age of 68.85 ± 11.22, and RATS had a mean age of 65.78 ± 17.50 (P=0.586). Operative time was statistically longer in the RATS group compared to the VATS group (VATS, 112.64 ± 37.64; RATS, 201.57 ± 87.87 (P=0.003)). Overall, there were no significant differences between complications, mortality, tumor size, and pathologic stage.

## Discussion

As surgical technology continues to advance, the landscape of thoracic surgery is also evolving accordingly. Ongoing developments in robotic and laparoscopic techniques, instrumentation, and training continue to refine the capabilities of both RATS and VATS.

VATS was first introduced in the early 1990s; it rapidly gained popularity due to less pain, shorter hospital stays, and decreased morbidity compared to traditional open thoracotomy [7]. Yet despite its clear advantages, VATS procedures have drawbacks such as restricted instrument mobility and lack of 3D perspective, which negatively impact depth perception. These contribute to difficulty in finer dissection and laparoscopic suturing [8]. These issues were alleviated somewhat by the advent of robotic surgery. The da Vinci robotic system was approved by the US Food and Drug Administration in 2000, and the first robotic thoracic surgery was reported to be done in the year 2001 [9]. Since then, robotic thoracic surgery has become widespread, in part due to advanced instrumentation that allows for seven degrees of freedom of motion and improved surgeon precision and ergonomics [10-12].

When we look at comparative studies between RATS and VATS, RATS has been shown to be as effective as, and potentially even superior to, VATS. Kent et al. analyzed a large database of pulmonary resections and found no statistically significant difference in mortality, length of stay, and overall complication rates between RATS and VATS. Interestingly, however, Kent et al. found that pulmonary resections performed with RATS had statistically significant decreased mortality, length of stay, and overall complications compared to open thoracotomy [13]. Furthermore, in a large retrospective study done by Li et al. comparing RATS to VATS lobectomies, they noted improved results for RATS regarding lymph node harvest, chest tube duration, and length of hospital stay. These results were all statistically significant [14]. Our study aims to provide additional insight into the feasibility and advantages of robotic surgery as compared to the current standard of care, which is VATS. 

This study reinforced that both RATS and VATS are viable approaches for thoracic surgical interventions, each offering distinct advantages and limitations. Regarding the operative time, the result of the meta-analysis revealed that the operative time was similar between the two groups, with no statistical difference, which was consistent with the results reported by Liang et al. [15]. However, most previous studies reported a longer operative time for RATS compared to VATS, which was contrary to our results [16]. Although operative times were longer with RATS, blood loss was significantly lower, and length of stay was significantly shorter. The precision, flexibility, and control offered with robotic-assisted surgery can lead to beneficial clinical outcomes that improve patients’ quality of life both in the immediate postoperative period as well as in their overall recovery from surgery. Despite the longer duration of time under general anesthesia, this study showed that patients who underwent RATS were able to be discharged home earlier than patients who underwent VATS. Both groups had similar rates of complications and 30-day mortality. There were no statistical differences in length of ICU stay or number of lymph nodes harvested.

Cost is not addressed in our study, but it is an important consideration when deciding between RATS and VATS. Current literature demonstrates that RATS is more expensive than VATS; the price of the robot itself, the necessary investment in robotic training, the disposable instruments that must be replaced, and robotic maintenance must all be included when calculating expenditure [17]. However, as institutional experience surrounding robotic thoracic surgery increases, costs will likely decrease with time. Studies suggest that the main contributing factor to the decreased cost of VATS compared to open thoracotomy is due to decreased length of stay. As data hve shown that length of stay is further decreased in RATS as compared to VATS, which may partially offset the increased cost associated with robotic surgery [18, 19], Future studies assessing RATS and VATS are recommended to focus on analyzing not only perioperative cost but also cost associated with total hospital stay to get a comprehensive picture of total cost.

Both VATS and RATS exhibit comparable safety profiles and similar overall patient outcomes. Given the decreased blood loss and shorter length of hospital stay, the RATS group has an advantage that may offset the longer operative time and increased cost associated with robotic surgery. It is essential to acknowledge that the choice between RATS and VATS is influenced by factors such as patient characteristics, surgeon expertise, procedural complexity, and institutional resources. The decision-making process should be guided by a thorough assessment of these variables to ensure that the optimal surgical approach is selected for each individual case. Conversion from RATS to VATS in the literature is low, and in our case, we had two conversions from RATS to open thoracotomy (5%) [20]. 

## Conclusions

RATS and VATS have distinct merits, and the choice between them should be based on a comprehensive assessment of various factors, including disease complexity, institutional resources, and long-term care goals. In conclusion, our analysis suggested that RATS is a feasible and safe technique that can achieve the same or even better surgical efficacy compared with VATS in terms of short-term and long-term outcomes. This study highlights the importance of advanced surgical techniques with individual patient needs. As robotic technology continues to advance and become more accessible, its role in thoracic surgery is likely to expand. Continued research and cost-effectiveness evaluations will be essential to guide these evolving techniques into standard practice.
